# Estimation of prevalence of sarcopenia by using a new bioelectrical impedance analysis in Chinese community-dwelling elderly people

**DOI:** 10.1186/s12877-016-0386-z

**Published:** 2016-12-28

**Authors:** Hui Wang, Shan Hai, Li Cao, Jianghua Zhou, Ping Liu, Bi-Rong Dong

**Affiliations:** 1 Center of Gerontology and Geriatrics, West China Hospital, Sichuan University, No. 37 Guoxue Lane, Chengdu, 610041 China; 2 School of Clinical Medicine, Chengdu Medical College, Chengdu, China; 3 Collaborative Innovation Center of Sichuan for Elderly Care and Health, Chengdu, China

**Keywords:** Sarcopenia, Elderly, Dual-energy X-ray absorptiometry, Bioelectrical impedance analysis, Appendicular skeletal muscle, Asian Working Group for Sarcopenia

## Abstract

**Background:**

The aim of the present study was to validate the usefulness of the new octapolar multifrequency bioelectrical impedance analysis (BIA) for assessment of appendicular skeletal muscle mass (ASM) by comparing it with that of dual-energy X-ray absorptiometry (DXA) and to investigate the prevalence of sarcopenia in Chinese community-dwelling elderly according to Asian Working Group for Sarcopenia (AWGS) definition.

**Methods:**

A cross-sectional study was conducted in communities of Chengdu, China. A total of 944 community-dwelling elderly adults aged ≥60 years were included. ASM was measured by using DXA as a criterion method to validate a standing eight-electrode multifrequency BIA (InBody 720), followed by a further estimation of the prevalence of sarcopenia according the AWGS definition.

**Results:**

In the Bland-Altman analysis, no significant difference was found between DXA and BIA based on the ASM measurements. The prevalence of AWGS-defined sarcopenia was 12.5% in the elderly women and 8.2% in the elderly men.

**Conclusions:**

BIA is suitable for body composition monitoring (ASM) in elderly Chinese as a fast, noninvasive, and convenient method; therefore, it may be a better choice in large epidemiological studies in the Chinese population. The prevalence of AWGS-defined sarcopenia was approximately 10.4% and increased with age in the Chinese community-dwelling elderly in this study.

## Background

Sarcopenia is the loss of muscle mass and function (defined by muscle strength or physical performance) with aging [1], which may result in reduced physical capability, quality of life, falls, disabilities, morbidities, and mortality, as well as high health care expenditure, in older people [2, 3]. The prevalence of sarcopenia has been reported to increase rapidly with aging [4, 5]. It was around 20% among people aged ≥65 years and may reach 50–60% among octogenarians [6]. As China is the most populated and fastest aging country in the world, sarcopenia poses a great impact on Chinese populations in the near future [7]. In 2010, the European Working Group on Sarcopenia in Older People (EWGSOP) proposed an operational definition and diagnostic strategy for sarcopenia that had become the most widely used in the world [8]. In 2014, the Asian Working Group for Sarcopenia (AWGS) also established the consensus on sarcopenia diagnosis, which recommended a set of approaches for the measurement of muscle mass, muscle strength, and physical performance, and chose different cutoff values according to the evidence derived from Asian populations. However, no epidemiological survey has used the AWGS definition to estimate the prevalence of sarcopenia in China.

According to the AWGS definition, the measurable variables include muscle mass, muscle strength (handgrip strength [HS]), and physical performance (gait speed [GS]). The challenge is to determine how best to measure them accurately, especially appendicular skeletal muscle mass (ASM). Several techniques have been used to assess ASM, including dual energy x-ray absorptiometry (DXA), computed tomography (CT), magnetic resonance imaging (MRI), and bioelectrical impedance analysis (BIA) [9]. Solid evidence from cadaver studies shows that DXA, MRI, and CT provide precise and reliable measurements of SM and thus can be considered as criterion methods for measuring ASM in vivo [10–12]. However, certain factors such as cost, accessibility, and the problem of radiation exposure limit the use of whole-body imaging. By contrast, BIA, which is based on measurements of tissue conductivity in the study of human body composition, is relatively simple, quick (takes only a few minutes), and noninvasive. Technological advances over the past decade include an increase in the number of contact electrodes from 4 to 8 [13, 14], and the use of multifrequency electrical levels to substitute the single-frequency BIA. By using low- and high-frequency electric currents, the multifrequency BIA methods successfully avoid the problems encountered in the use of primal BIA devices. The different currents allow for the estimation of extracellular and intracellular water, as well as tolal body water [15].

Previous studies verified that compared with the DXA method, multifrequency and eight-electrode BIA provides reliable measurements of body composition in healthy subjects and in patients with stable water levels [16, 17]. InBody 720 (Biospace, Korea) is a new multifrequency analyzer with 4 pairs of electrodes (octapolar technology) embedded into the handles (thumb and palm electrodes) and floor scale (ball of foot and heel electrodes) of the analyzer. The researcher does not need to standardize the subject’s posture before BIA, which has the potential to reduce measurement times and makes this instrument ideal for epidemiological studies. Some previous studies evaluated the accuracy of InBody 720 with different reference methods or in different populations [18–20], but InBody 720 has not been validated for the estimation of ASM in Chinese populations.

The first purpose of this study was to validate the usefulness of InBody 720 for ASM by comparing it with DXA. Its second purpose was to investigate the prevalence of sarcopenia according to the AWGS definition in Chinese community-dwelling elderly.

## Methods

### Design

A cross-sectional survey to determine the prevalence of sarcopenia according to the AWGS definition in a Chinese community was conducted in two steps. First, 90 elderly volunteers (validation group) were recruited from 3 communities in Chengdu City to undergo BIA and DXA measurements to validate the accuracy of BIA for estimating ASM. Second, another 854 volunteers were recruited from the same communities with the same inclusive criterion, and the prevalence of sarcopenia in elderly people living in the community was investigated. The study staff was well trained in using investigation manuals, multimedia materials, and simulated patients. The trained interviewers collected the data from all the study participants at community centers by using face-to-face interviews. In the validation group, the BIA and DXA measurements were performed within 1–3 days. All the subjects refrained from alcohol intake for at least 48 h, from vigorous exercise for at least 12 h, and from taking a meal or drink for at least 12 h.

### Participants

We recruited 944 subjects (462 men and 482 women) aged 60 to 92 years who voluntarily participated in the study, through leaflets and posters provided by the Center of Gerontology and Geriatrics, West China Hospital, Sichuan University, between March 2014 and October 2014. Volunteers were excluded if they had any diseases such as hyperthyroidism or hypothyroidism, or chronic heart and renal failure, or been receiving prescribed medications such as long-term steroid treatment, which is known to affect body composition. Individuals who could not communicate with the interviewers owing to severe cognitive impairment, mental disorders, and severe hearing and eye problems were also excluded from the study. All the participants were ambulatory without physical disability or amputation. The study was approved by the ethics committee of Sichuan University, under reference No. 2014 (57). Written consent was obtained from each participating subject prior to testing.

### Anthropometric measurements

Each subject was interviewed by using a structured questionnaire to obtain basic demographic data and information on medical conditions. Weight and height were measured while barefoot and wearing light clothing. Body weight was measured to the nearest 0.1 kg. Height was measured with a hypsometer to the nearest 0.1 cm. Body mass index (BMI) was calculated as weight in kilograms divided by height in meters squared.

### Muscle strength and gait speed

Muscle strength was assessed based on HS, measured by using a dynamometer (CAMRY EH101, CHINA). The participants were asked to exert maximum effort in a standing position, three readings were taken from each side, and the maximum value from the dominant hand was used for the analysis. Low handgrip strength was defined as <26 kg for men and <18 kg for women. The usual gait speed (m/s) on a 6-m course was used as an objective measure of physical performance. The participants were asked to walk 6 m at their usual pace, and the time required to walk the distance was measured to calculate gait speed (m/s). Use of a cane or walker was permitted if the participants could not perform the gait test without it. The gait test was performed twice, and the mean value was used in the analysis. Slow walking speed was defined as a walking speed of ≤ 0.8 m/s.

### Dual-energy X-ray absorptiometry

All the measurements were performed after overnight fasting and abstinence from alcohol and moderate to intensive exercise for more than 8 h. The participants in validation group performed anthropometric measurements, DXA, and BIA on the same day. Each participant was scanned by using DXA (iDXA GE, USA) for measuring appendicular muscle mass. For the imaging examination, the participants wore cotton clothing without any metal attachments. Scans were performed in the whole-body scan mode in the following order: head, upper limbs, lower limbs, and trunk. Each participant underwent approximately 20 min of whole-body scan. Results were analyzed with software (12.10.017, GE, US).

### Bioelectrical impedance analysis

Bioelectrical resistance was measured by using Inbody720 (Biospace, Korea) at frequencies of 5, 50, 250, and 500 kHz. This instrument uses eight tactile electrodes, with four in contact with the palm and thumb of both hands and the other four in contact with the anterior and posterior aspects of the sole of both feet. The subject stands with the soles in contact with the foot electrodes and grabs the hand electrodes. The sequence of the measurements, controlled by a microprocessor, reports on the screen and paper. No precaution was taken to standardize the subject’s posture before BIA, as suggested by the manufacturer. RI values were calculated at all frequencies. Data output, as calculated by using the manufacturer’s algorithm, included fat mass and skeletal muscle mass of the total body, arms, and legs.

### Diagnosis of sarcopenia

Sarcopenia was defined according to the AWGS algorithm, in which the patient has low muscle mass, and low muscle strength or low physical performance. As suggested by the AWGS, low muscle mass was defined as an ASM index (ASMI, ASM/height^2^) of <7.0 kg/m^2^ in men and <5.7 kg/m^2^ in women. Low muscle strength was defined as a handgrip strength of <26 kg in men and <18 kg in women; and low physical performance, as a gait speed of <0.8 m/s.

### Statistical analyses

All statistical analyses were performed by using SPSS version18.0 for Windows (IBM Corp, Armonk, NY). Baseline characteristics were compared between the men and women in the validated and elderly groups by using an independent-samples *t* test for continuous variables and the Pearson chi-square test or Fisher exact test (for which an expected cell count was <5) for categorical variables. Pearson correlations were performed to examine the relationship between the DXA- and the InBody 720-measured ASMs. Bland-Altman analyses were performed to evaluate the extent of agreement between both methods. The differences in the continuous variables were compared by using one-way analysis of variance (ANOVA). Frequency data were compared by using the Pearson chi-square test or Fisher exact test (for which an expected cell count was <5). Differences were considered significant at *p* < 0.05.

## Results

### The characteristics of the subjects

Table 1 lists the characteristics of the elderly participants according to sex. The mean age and BMI in the validated and elderly groups were respectively 68.71 ± 6.60 and 68.76 ± 6.65 years, and 24.27 ± 3.25 and 23.88 ± 3.05 (*p* > 0.1). We did not find significant sex-related differences in BMI and age. Although the weight and BMI in the elderly male group were smaller than those in the validated group (*p* < 0.05), the differences were not clinically significant. The sex-related differences in each anthropometric variable (expect BMI) were significant in both the validated and older groups. The men were taller and heavier, and had higher ASM and ASMI (*p* < 0.001). For physical performance, the men had significant stronger HS in both groups (35.50 ± 7.38 vs. 23.29 ± 4.12 and 36.58 ± 7.08 vs. 23.47 ± 4.58, respectively; *p* < 0.001) and walked a little faster than the women in the elderly group (1.08 ± 0.20 vs. 1.02 ± 0.18, *p* < 0.001). The percentages of current alcohol drinkers and smokers were 42.4 and 28.0% in the men, and 12.5 and 2.7% in the women, respectively. The most prevalent medical diseases were hypertension (43.5%) and diabetes (19.0%). No significant sex-related difference or differences between the validated and elderly groups were observed. The incidence rates of the remaining medical diseases were all <5%.

**Table 1:** Comparison of baseline characteristics between the validated group and the elderly group

		Validated group		Elderly group		*p* value
		n	mean ± SD	n	mean ± SD	
Age (year)	M	46	69.85 ± 7.03	419	69.10 ± 6.59	0.267
	F	47	67.89 ± 6.11	435	68.52 ± 6.44	0.524
Height (cm)	M	43	163.85 ± 6.30	419	164.14 ± 6.16	0.770
	F	47	153.00 ± 5.76	435	152.56 ± 5.99	0.636
Weight (kg)	M	43	67.14 ± 10.70	419	64.01 ± 9.16	0.037
	F	47	55.32 ± 7.70	435	56.02 ± 8.64	0.594
BMI (kg/m^2^)	M	43	24.93 ± 3.11	419	23.72 ± 2.83	0.009
	F	47	23.66 ± 3.28	435	24.04 ± 3.25	0.451
GS (m/s)	M	43	1.07 ± 0.19	418	1.08 ± 0.20	0.827
	F	47	1.03 ± 0.15	435	1.02 ± 0.18	0.708
HS (kg)	M	43	35.50 ± 7.38	419	36.58 ± 7.08	0.346
	F	47	23.29 ± 4.12	435	23.47 ± 4.58	0.792
ASM (kg)						
BIA	M	43	19.72 ± 2.85	419	19.63 ± 2.95	0.830
	F	47	13.86 ± 2.25	435	14.02 ± 2.36	0.667
DEXA	M	43	18.74 ± 3.16	0	*	
	F	47	13.37 ± 2.13	0	*	
ASMI (kg/m^2^)						
BIA	M	43	7.32 ± 0.69	419	7.26 ± 0.75	0.591
	F	47	5.89 ± 0.66	435	5.99 ± 0.72	0.379
DEXA	M	43	6.95 ± 0.87	0	*	
	F	47	5.70 ± 0.77	0	*	
Medical diagnoses						
Hypertension	M	43	19 (44.2)	419	178 (42.5)	0.830
	F	47	18 (38.3)	435	196 (45.2)	0.368
Diabetes	M	43	6 (14.0)	419	82 (19.6)	0.372
	F	47	10 (21.3)	435	82 (18.9)	0.688
Thyroid diseases	M	43	0	419	2 (0.5)	1.000
	F	47	0	435	4 (0.9)	1.000
Cardiac diseases	M	43	1 (2.3)	419	23 (5.5)	0.373
	F	47	7 (14.9)	435	20 (4.6)	0.004
Renal diseases	M	43	0	419	6 (1.4)	1.000
	F	47	2 (4.3)	435	7 (1.6)	0.216
Stroke	M	43	0	418	4 (1.0)	1.000
	F	47	0	434	5 (1.2)	1.000
Cancer	M	43	1 (2.3)	419	3 (0.7)	0.324
	F	47	0	435	2 (0.5)	1.000
COPD	M	43	0	419	5 (1.2)	1.000
	F	47	1 (2.1)	435	7 (1.6)	0.563
Tuberculosis	M	43	0	419	9 (2.1)	1.000
	F	47	0	435	1 (0.2)	1.000
Hepatic diseases	M	43	1 (2.3)	419	8 (1.9)	0.588
	F	47	0	435	7 (1.6)	1.000
Smoking habits	M	43	12 (27.9)	419	118 (28.2)	0.972
	F	47	0	435	13 (3.0)	0.626
Alcohol consumption	M	43	24 (55.8)	419	173 (41.3)	0.067
	F	47	12 (25.5)	435	48 (11.1)	0.004

*: no data; *BMI* body mass index, *GS* gait speed, *HS* handgrip strength, *ASM* appendicular skeletal muscle mass, *ASMI* appendicular skeletal muscle mass index, *BIA* bioelectrical impedance analysis, *DXA* dual energy x-ray absorptiometry, *COPD* chronic obstructive pulmonary disease; Using independent-samples *t* test for continuous variables and Pearson chi-square or Fisher exact test (where an expected cell count was < 5) for categorical variables. During testing, *p* < 0.05 was considered statistically significant

### Correlation between BIA- and DXA-measured ASM

**Fig. 1:**
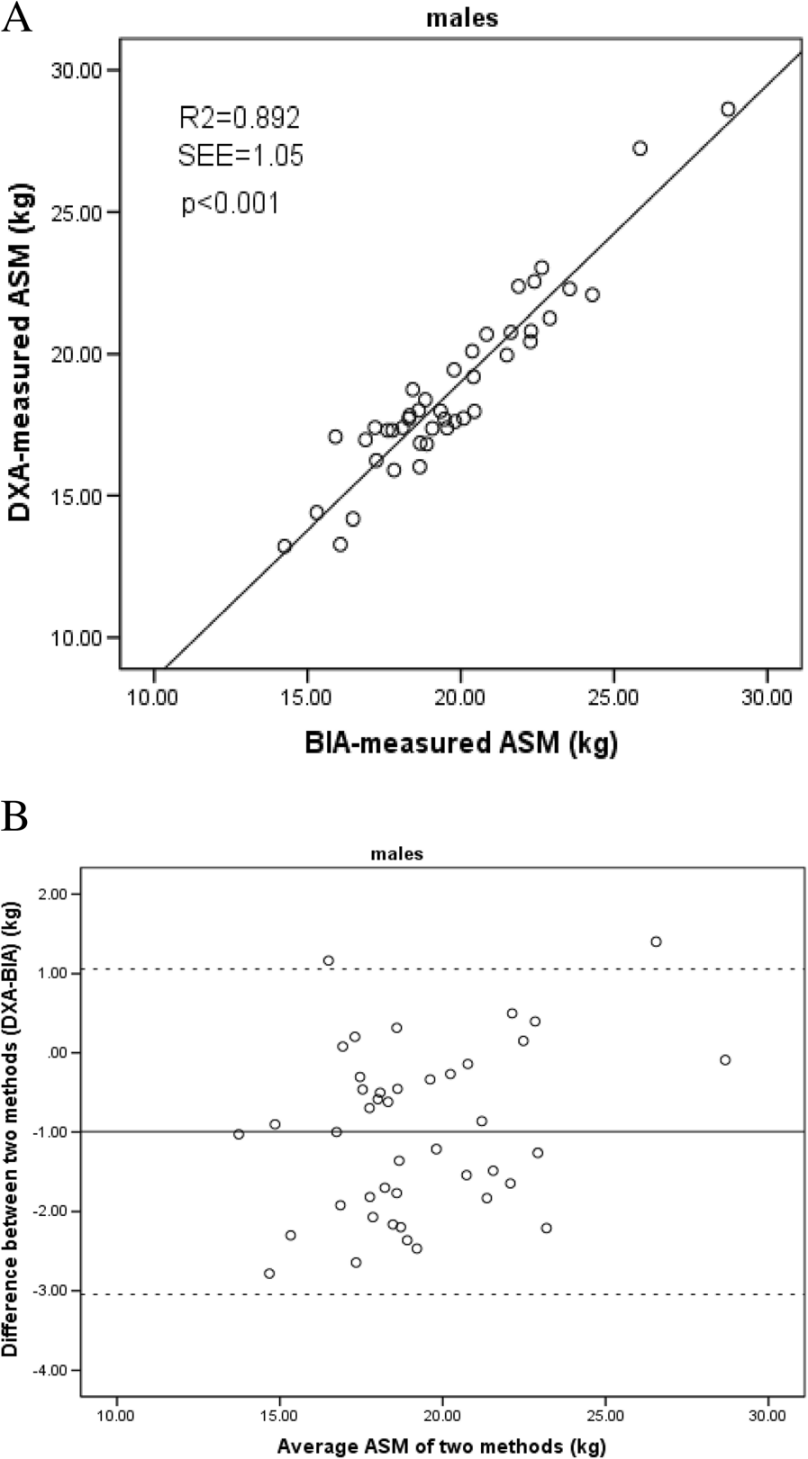
**a** Regression between the bioelectrical impedance analysis (BIA)-measured and Dual-energy X-ray absorptiometry (DXA)-measured appendicular skeletal muscle mass (ASM) in males. Solid line, regression line. **b** Bland-Altman plot for difference between DXA-measured and BIA-measured ASM and the average ASM of the two methods in males. Solid line represents the mean difference (−0.99 kg); Outer dotted lines represent limits of agreement (−3.05 to 1.06 kg) (95% confidence interval). *r* ^2^ = coefficient of determination; SEE = standard error of the estimate

**Fig. 2:**
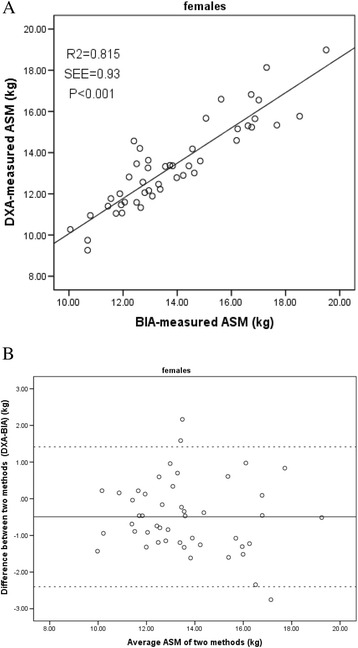
**a** Regression between the bioelectrical impedance analysis (BIA)-measured and Dual-energy X-ray absorptiometry (DXA)-measured appendicular skeletal muscle mass (ASM) in females. Solid line, regression line. **b** Bland-Altman plot for difference between DXA-measured and BIA-measured ASM and the average ASM of the two methods in females. Solid line represents the mean difference (−0.49 kg); Outer dotted lines represent limits of agreement (−2.40 to 1.41 kg) (95% confidence interval). *r* ^2^ = coefficient of determination; SEE = standard error of the estimate

The correlation between ASM obtained by using BIA and DXA was high in both the men and women (Figs. 1a and 2a). The Pearson correlation coefficient and standard error of the estimate (SEE) of the regression equation were 0.94 and 1.05 kg in the men, and 0.90 and 0.93 kg in the women, respectively (both *p* > 0.05). No significant method-related biases were found. The agreement of these two methods in the Bland-Altman analysis is presented in Figs. 1b and 2b. Only 2 points were outside the limit of agreement in the men; and 3 points, in the women.

### Differences in body composition, muscle strength, and physical function according to age and sex

The differences in body composition, physical functions (represented by GS), and muscle strength (represented by HS) according to age and sex are shown in Table 2. ASM, ASMI, GS, and HS all showed significant declines with age in both sexes (*p* < 0.005).

**Table 2:** Differences in body composition, muscle strength and physical function with age and gender

	Age group (year)	Number	ASM (kg)	ASMI (kg/m2)	HS (kg)	GS (s)
Males	60–64	146	20.24 ± 0.26	7.43 ± 0.06	39.62 ± 0.54	1.12 ± 0.02
	65–74	200	19.55 ± 0.20	7.23 ± 0.05	36.64 ± 0.46	1.09 ± 0.01
	≥75	117	19.03 ± 0.27	7.09 ± 0.07	32.21 ± 0.63	0.99 ± 0.02
*p*			0.003	0.001	<0.001	<0.001
Females	60–64	162	14.55 ± 0.18	6.10 ± 0.06	25.06 ± 0.35	1.08 ± 0.01
	65–74	226	14.13 ± 0.15	6.03 ± 0.05	23.40 ± 0.28	1.02 ± 0.01
	≥75	94	12.76 ± 0.22	5.67 ± 0.07	20.80 ± 0.44	0.92 ± 0.02
*p*			<0.001	<0.001	<0.001	<0.001

*ASM* appendicular skeletal muscle mass, *ASMI* appendicular skeletal muscle mass index, *HS* handgrip strength, *GS* gait speed, Using one-way ANOVA for continuous variables. During testing, *p* < 0.05 was considered statistically significant

### Estimated prevalence of low SM, low HS, low GS, and sarcopenia in the different age groups and the elderly group

The estimated prevalence of low SM, low HS, low GS, and sarcopenia in the different age groups and the elderly group are shown in Table 3. The criteria, including low SM, low HS, low GS, and sarcopenia, were defined by the AWGS [2]. The prevalence rates of low SM, low HS, and low GS increased significantly with age (*p* < 0.001). When the AWGS criteria were used to define sarcopenia, the prevalence rates of sarcopenia were 2.3, 5.6, and 19.9% in all the participants aged 60–64, 65–74, and ≥75 years, respectively (*p* < 0.001), and the age-related trend was also apparent in both sexes (*p* < 0.001). The prevalence rates of sarcopenia were 8.2, 12.5, and 10.4% in the elderly women, men, and both sexes, respectively.

**Table 3:** Estimated prevalence of low SM, low HS, low GS and sarcopenia in different age groups and the elderly

	Age group (year)	Number	Low muscle mass	Low gait speed	Low handgrip strenth	Sarcopenia
Male	60–64	146	40 (27.4)	4 (2.7)	5 (3.4)	4 (2.7)
	65–74	199	68 (34.2)	9 (4.5)	4 (2.0)	8 (4.0)
	≥75	117	52 (44.4)	18 (15.4)	20 (17.1)	18 (15.4)
	elderly	316	120 (38.0)	27 (8.6)	24 (7.6)	26 (8.2)
*p*			0.015	<0.001	<0.001	<0.001
Female	60–64	162	20 (12.3)	6 (3.7)	6 (3.7)	3 (1.9)
	65–74	226	37 (16.4)	17 (7.5)	17 (7.5)	16 (7.1)
	≥75	94	36 (38.3)	22 (23.4)	25 (26.2)	24 (25.5)
	elderly	320	73 (22.8)	39 (12.2)	42 (13.1)	40 (12.5)
*p*			<0.001	<0.001	<0.001	<0.001

Low muscle mass was defined as appendicular skeletal muscle mass index (ASMI) <7 kg/m2 for males and <5.7 kg/m2 for females; low gait speed (GS) as GS <0.8 m/s; low handgrip strength (HS) as HS <26 kg for males or <18 kg for females. Using Pearson chi-Square tests or Fisher exact test (for which an expected cell count was <5) for categorical variables. Comparison between 60-64, 65-74, and ≥75, *p* < 0.05 was considered statistically significant

## Discussion

MRI, CT, and DXA measurements of ASM are not suitable for large-scale surveys on sarcopenia in the elderly due to cost, accessibility, and the problem of radiation exposure. BIA might be a good alternative method to measure ASM for epidemiological surveys. However, a previous study reported that BIA equations chosen should not be used without prior verification against reference methods in the subject population studied because of existing differences in body build among ethnic groups [21]. To the best of our knowledge, this is the first study that validated the usefulness of a multifrequency octapolar BIA, InBody 720, and its predictive ability for estimating ASM by comparing it with those of DXA in community-dwelling elderly people in China. The results of the present study demonstrated the Pearson correlation coefficients (0.944 and 0.903) and SEE (1.051 and 0.927 kg) in men and women, respectively, in predicting ASM, which were similar to those obtained in previous reports [16, 22, 23]. Houtkouper et al. suggested that prediction errors (SEE) of 2.0–2.5 kg in men and 1.5–1.8 kg in women are considered ideal [21]. Although the negative slope of the Bland-Altman plot indicated a trend of underestimation and overestimation errors with the BIA method for participants with lower and higher ASMs, respectively, the mean error was small and insignificant. Thus, this result suggests that the BIA method provided a meaningful ASM estimation in the Chinese populations. However, our observation conflicts with the results of Anderson et al., who found a significant InBody 720 underestimation of ASM in men (−3.0 kg) and women (−1.0 kg) [24]. Their study population was quite different from ours; their subjects were not Asians, were aged 18 to 49 years, and had slightly higher BMIs (25.8 ± 4.5 kg/m^2^). Another study conducted in a Finnish population found that compared with DXA, InBody 720 provided on average 2–6% lower values for fat mass (FM) in men with normal BMI and in all BMI categories in women. However, the authors did not report the correlation of ASM. But in our recent research, we did not validate the correlation according to BMI categories.

Association of sarcopenia with poor health status and adverse outcomes had triggered a new approach for health promotion and health care of older people. Nutrition and exercise interventions can help prevent age-related decline in physical performance and increase in mortality [25, 26]. Although the prevalence of sarcopenia in elderly people has been broadly investigated in Asia, the large variability in prevalence is partly related to the differences in measurements and cutoffs used to define sarcopenia. Hence, the AWGS developed a consensus definition and cutoff points for sarcopenia in Asia. To our knowledge, this is the first large epidemiological survey that used the AWGS definition to estimate the prevalence of sarcopenia in Chinese community-dwelling elderly. The results show prevalence rates of 12.5% and 8.2% in women and men, respectively. The prevalence of sarcopenia varies across studies because of different study populations, ages, sex, and diagnosis criteria [25]. Focusing on the Chinese elderly, between-study variations in the prevalence of sarcopenia also exists. We found only 3 studies that used the AWGS definition in Chinese elderly. One study is currently conducted in Chinese suburb-dwelling elderly, and the authors reported that the prevalence of sarcopenia was 11.5% in women and 6.4% in men [27], which were similar to the outcomes in our study. Another research study found that the prevalence rates of sarcopenia (using the AWGS definition) were 5.9% in men and 0.7% in women [18], which were much lower than ours. Their research only included 286 participants with a mean age of 66.5 ± 4.8 years in men and 65.4 ± 4.5 years in women. The difference in age partly resulted in the diversity of prevalence. Another study reported that the prevalence rates of AWGS-defined sarcopenia were 7.0 and 13.1% in Chinese urban and rural elderly, respectively. These values indicate a significant difference, which was partly due to underdevelopment of living and socioeconomic conditions in rural areas. However, the researchers applied only anthropometric measures for estimating skeletal muscle mass instead of BIA or DXA [28]. Some studies also investigated the prevalence of sarcopenia in Chinese populations by using different diagnostic criteria. According to a large cohort study involving 4000 Chinese community-dwelling adults aged ≥65 years in Hong Kong, the prevalence of sarcopenia (defined by the EWGSOP criteria) was 9% at baseline [29], which was similar to the outcome in our study. However, a survey in Taiwan found that the prevalence (defined by a BMI of 8.87 kg/m^2^ in men and 6.42 kg/m^2^ in women) of sarcopenia was 18.6% in women and 23.6% in men, which were much higher than our results. It also reported a great discrepancy in prevalence of sarcopenia, from 14 to 37% in women and from 13 to 42% in men, in the same sample depending on the definition used [5]. The tremendous variations within the same population shown in the previous research studies bring into question the results of the existing studies on sarcopenia prevalence. These variations were partly due to the different participants and definitions of sarcopenia. Furthermore, the appropriate definition of sarcopenia in Chinese older adults remains unclear [30, 31]. Therefore, large population-based perspective surveys are required for Chinese people to establish ethnic-specific references and cutoff values for older adults [32].

Similar to those of other authors [4, 30, 33, 34], our present findings also confirmed the decrease in ASM, ASMI, HS, and GS, and the increase in the prevalence rates of low muscle mass, low muscle strength, and low physical performance with age. We observed that ASM was significantly lower among the women aged ≥75 years than among those in the preceding age group (12.76 ± 0.22 kg vs. 14.13 ± 0.15 kg, *p* < 0.001). Moreover, even though ASM decreased dramatically, body weight just decreased slightly with aging, partly due to the increase in FM (unreported data). The decrease in ASM and increase in FM accompanying aging may lead to the development of sarcopenia and contribute to obesity in the elderly [33]. We also found that muscle strength (HS) and physical performance (GS) decreased more greatly with age than did muscle mass (*p* < 0.001), which was similar to the findings of previous cross-sectional or longitudinal investigations [30, 35–38]. Goodpaster found that even maintenance or gain of lean mass in the elderly did not prevent loss of strength [39]. Despite extensive research efforts, the mechanism responsible for the age-associated decline in muscle strength has not been completely elucidated, but the age-associated decline in muscle mass certainly plays a partial role [38]. Muscle strength, rather than muscle mass, is more important in estimating adverse outcomes such as physical disability, poor quality of life, and higher mortality [39–41]. For this reason, both EWGS and AWGS recommended the use of both low muscle mass and function (strength or performance) for the diagnosis of sarcopenia [2, 8].

### Study limitations

Some limitations of this study should be pointed out. First, as previously mentioned, nonrandom selection of the study population and the exclusion of elderly people with severe chronic diseases from our study group might have resulted in a selection bias. Consequently, the study group, which included individuals who were relatively healthy and had good activity level, might have caused, to some extent, the underestimation of the prevalence of sarcopenia. Second, we used DXA as the “gold standard” reference method to validate BIA (InBody 720). Although validation in comparison with DXA is the most feasible and appropriate choice for our research, it is possibly not the most accurate analysis. The sample sizes for the Bland-Altman analysis should be more than 100; however, in our study, we only collected 90 participants because of the limited condition. In our future research, we should expand the sample sizes to decrease the bias. Finally, as a cross-sectional study, it may not provide the same results as a longitudinal or semi-longitudinal study. This paper presents the first-stage research results. In the next-stage research, the change trend of muscle mass, muscle strength, physical performance, and other outcome indicators such as falls, hospital admission, and mortality will be investigated.

## Conclusion

In conclusion, the relatively high correlation coefficient between the BIA- (InBody 720) and DXA-measured ASMs indicates that the application is suitable for body composition monitoring in elderly Chinese in a fast, noninvasive, and convenient way. The study also demonstrates that the prevalence of AWGS-defined sarcopenia was approximately 10.4% and increased with age in a sample of Chinese community-dwelling elderly.

## Abbreviations

ASM: Appendicular skeletal muscle mass
ASMI: Appendicular skeletal muscle mass index
AWGS: Asian Working Group for Sarcopenia
BIA: Bioelectrical impedance analysis
BMI: Body mass index
CT: Computed tomography
DXA: Dual-energy X-ray absorptiometry
EWGSOP: European Working Group on Sarcopenia in Older People
FM: Fat mass
GS: Gait speed
HS: Handgrip strength
MRI: Magnetic resonance imaging
SEE: Standard error of the estimate
